# Chemical Composition, Antioxidative and Anticancer Activities of the Essential Oil: *Curcumae Rhizoma*–*Sparganii Rhizoma*, a Traditional Herb Pair

**DOI:** 10.3390/molecules200915781

**Published:** 2015-08-28

**Authors:** Guan-Ling Xu, Di Geng, Meng Xie, Kai-Yue Teng, Yu-Xin Tian, Zi-Zhen Liu, Cheng Yan, Yan Wang, Xia Zhang, Yan Song, Yue Yang, Gai-Mei She

**Affiliations:** 1 School of Chinese Pharmacy, Beijing University of Chinese Medicine, Beijing 100102, China; E-Mails: xuguanling4004@163.com (G.-L.X.); piece124@sohu.com (D.G.); xiemeng0819@163.com (M.X.); teng1995815@163.com (K.-Y.T.); tianyuxin1216@163.com (Y.-X.T.); lzz332@126.com (Z.-Z.L.); 13269650940@163.com (C.Y.); 18739908461@163.com (Y.W.); zhangxia0561@126.com (X.Z.); 18311242015@163.com (Y.Y.); 2 Pharmacy College, Ningxia Medical University, Ningxia 750021, China; E-Mail: songyan200714@163.com

**Keywords:** *Curcumae Rhizoma*, *Sparganii Rhizoma*, *Curcumae Rhizoma*–*Sparganii Rhizoma*, herb pair, essential oils, antioxidant activity, anti-cancer activity

## Abstract

As a classical herb pair in clinics of traditional Chinese medicine, *Curcumae Rhizoma*–*Sparganii Rhizoma* (HP CR–SR) is used for activating blood circulation to remove blood stasis. The essential components in HP CR–SR and its single herbs were comparatively analyzed using gas chromatography-mass spectrometry data. 66, 22, and 54 components in volatile oils of *Curcumae Rhizoma*, *Sparganii Rhizoma*, and HP CR–SR were identified, and total contents accounted for 75.416%, 91.857%, and 79.553% respectively. The thirty-eight components were found in HP CR–SR, and not detected in single herbs *Curcumae Rhizoma* and *Sparganii Rhizoma*. The highest radical trapping action was seen by an essential oil of HP CR–SR (IC_50_ = 0.59 ± 0.04 mg/mL). Furthermore, the HP CR–SR essential oil showed more remarkable cytotoxicity on tumor cell lines than that of the single herbs *Curcumae Rhizoma* and *Sparganii Rhizoma* in a dose-dependent manner: IC_50_ values showing 32.32 ± 5.31 μg/mL (HeLa), 34.76 ± 1.82 μg/mL (BGC823), 74.84 ± 1.66 μg/mL (MCF-7), 66.12 ± 11.23 μg/mL (SKOV3), and 708.24 ± 943.91 μg/mL (A549), respectively. In summary, the essential oil of HP CR–SR is different from any one of *Curcumae Rhizoma* and *Sparganii Rhizoma*, nor simply their superposition, and HP CR–SR oil presented more remarkable anticancer and antioxidant activities compared with *Curcumae Rhizoma* and *Sparganii Rhizoma* oils.

## 1. Introduction

Herbal pair (HP), as a basic unit in compatibility of traditional Chinese medicine (TCM), has been widely applied in folk medicine for thousands of years [1,2]. Based on the compatibility mechanism, HP was composed of two relatively fixed herbs in the clinic. When the two fixed herbs are used together, interactions will occur so as to enhance the effect, reduce the toxicity or even remove the side effects compared with using a single herb alone [3,4,5]. It has been proved that HP played an important role in the development of TCM [2]. Compared with TCM compatibility, HP is not only based on the basic features and principles of TCM compatibility, but also easier to study due to their simpler chemical compositions [2]. Therefore, the studies to HP chemistry will provide a different and relatively simple way for the study of prescription chemistry in TCM.

*Curcumae Rhizoma*–*Sparganii Rhizoma* (CR–SR) is a classical herbal pair in clinics. It is used for activating blood circulation to remove blood stasis. CR is good at breaking the Qi and dispersing the blood accumulation; SR is expert in removing blood stasis and dredging meridians [6]. HP CR–SR was first recorded as Sanleng Wan in “*Jingyan Liangfang*” during the Qing dynasty, and has long been applied for the treatment of blood stasis and dysmenorrheal from the TCM perspective [7,8]. So far, HP CR–SR has been involved as a TCM prescription preparation in Chinese pharmacopoeia, such as Fuke Tongjing Wan, Tongjingbao Keli, commonly being used to cure dysmenorrheal [9]. Moreover, HP CR–SR was usually used to treat tumors in the TCM clinic, especially with respect to the gynecology aspect [10,11,12].

At present, modern pharmacological research and clinical practices mostly focus on nonvolatile fractions of HP CR–SR, and it possesses extensive activities, including anti-cancer activity [13,14,15,16], anti-inflammatory activity, and anti-thrombotic activity [17]. The pharmacological activities of HP CR–SR essential oil are rarely reported and there is only one paper on the chemical components of HP CR–SR oil [18]. It is noticed that CR essential oil has been recorded in the Pharmacopoeia of People’s Republic of China (2010 edition), to treat gynecological oncology [9,19,20,21]. The potential biological damage caused by free radicals is a leading contributor to chronic diseases, including cancer, diabetes mellitus, aging, and neurodegenerative disorders [22,23] and the essential oil of CR was also good at antioxidant activity [24,25]. The difference between the HP oil and CR oil has not been described from the perspective of chemical compositions and pharmacological activities to the best of our knowledge.

In this paper, the essential oils from CR, SR, and HP CR–SR have been extracted, respectively, and the components have been identified by gas chromatography-mass spectrometry (GC-MS). Meanwhile, their antioxidant activity was assessed by scavenging free radical ability, and antitumor activity, *in* *vitro*, was also evaluated with several cell models by MTT assay. The aim of this work was to clarify the compatibility of HP CR–SR in the TCM clinic from the view of chemical compositions and the pharmacological activities.

## 2. Results and Discussion

Zedoary turmeric oil, the essential oil extracted from CR, is documented in the Chinese Pharmacopoeia (2010) that it has exhibited many potential medical characteristics in the aspects of anticancer and antivirus [26,27]. In records of Chinese medical formula books or traditional application, CR is always combined with SR [7,8]. The difference between HP oil and CR oil has been analyzed by GC-MS.

### 2.1. The Volatile Components of CR, SR and HP CR–SR

The essential oils of CR, SR, and HP CR–SR were extracted by hydrodistillation, and the yield was 6.0 mL/kg, 0.1 mL/kg, and 2.8 mL/kg, respectively. By means of the overall volume integration method, quantitative results of each component in pure chromatographic profiles of HP CR–SR, single herbs CR and SR can be obtained, as shown in Table 1. In total, 66, 22, and 54 volatile components in CR, SR, and HP CR–SR were determined quantitatively, accounting for 75.416%, 91.857%, and 79.553%, respectively. The volatile chemical components in HP CR–SR are notably different from those of single herb CR or SR.

**Table 1:** Main chemical components of volatile oils from CR, SR, and HP CR–SR.

No.	Name of Components	RI	CAS Identification Number	CR		SR		HP (CR–SR)	
				φ/%	t/min	φ/%	t/min	φ/%	t/min
1	4-Hydroxy-4-methyl-2-pentanone	834	123-42-2	-	-	5.691	4.487	4.343	4.490
2	Isoborneol	1163	124-76-5	0.098	13.087	-	-	-	-
3	Borneol	1172	507-70-0	0.229	13.327	-	-	-	-
4	α-Terpineol	1194	98-55-5	0.263	13.970	-	-	-	-
5	(2*E*,4*E*)-Decadienol	1300	18409-21-7	0.023	16.927	-	-	-	-
6	Dimethoxy-(*Z*)-Citral	1310	75128-97-1	0.042	17.168	-	-	-	-
7	Geranyl acclate	1355	105-87-3	0.025	18.379	-	-	-	-
8	*neiso*-Dihydro carveol acetate	1360	256332-36-2	0.021	18.495	-	-	-	-
9	(*Z*)-Trimenal	1371	300733-87-3	0.012	18.794	-	-	-	-
10	*trans*-Menth-6-en-2,8-diol	1379	42370-41-2	0.008	19.014	-	-	-	-
11	Orcinol	1382	504-15-4	-	-	0.008	19.090	-	-
12	β-Elemene	1389	515-13-9	0.089	19.280	-	-	-	-
13	Myltayl-(12)-ene	1455	79562-97-3	0.286	20.937	-	-	-	-
14	Ethyl-(2*E*,4*Z*)-decadienoate	1466	3025-30-7	0.760	21.285	-	-	-	-
15	β-Chamigrene	1473	18431-82-8	0.406	21.405	-	-	-	-
16	α-Amorphen	1479	20085-19-2	0.278	21.550	-	-	-	-
17	α-Cuprenene	1480	29621-78-1	-	-	0.011	21.576	-	-
18	Isomethyl-α-(*E*)-Ionone	1488	55093-42-0	0.169	21.708	-	-	-	-
19	Premnaspirodiene	1490	82189-85-3	0.896	21.813	-	-	-	-
20	*cis*-β-Gualene	1493	88-84-6	0.096	21.896	-	-	-	-
21	α-Sclinene	1497	473-13-2	0.737	21.998	-	-	-	-
22	Lilial	1503	80-54-6	-	-	0.025	22.148	-	-
23	γ-Cadinene	1521	39029-41-9	0.265	22.561	-	-	-	-
24 *	Silphiperfol-5-en-3-one B	1545	199014-11-4	-	-	-	-	0.113	23.148
25 *	Elemd	1548	633-99-6	-	-	-	-	0.117	23.204
26	Hedycaryol	1548	21657-90-9	0.483	23.204	-	-	-	-
27	*trans*-Dauca-4(11),7-diene	1556	000-00-0	0.165	23.405	-	-	0.024	23.321
28 *	Germacrene B	1556	15423-57-1	-	-	-	-	0.105	23.400
29	Dodecanoic	1556	143-07-7	-	-	0.049	23.415	-	-
30	(*E*)-Nerolidol	1559	40716-66-3	-	-	0.014	23.486	-	-
31 *	Eremophila ketone	1561	158930-41-7	-	-	-	-	0.177	23.523
32	Longicamphenylone	1563	58560-59-1	0.256	23.526	-	-	-	-
33	Germacrene D-4-ol	1572	74841-87-5	0.125	23.787	-	-	-	-
34	Caryophyllenyl alcohol	1567	000-00-0	0.436	23.882	-	-	-	-
35 *	Caryolan-8-ol	1576	178737-45-6	-	-	-	-	0.144	23.890
36 *	Thujopsan-2-β-ol	1588	150737-93-2	-	-	-	-	0.132	24.033
37	Fokiend	1588	33440-00-5	0.586	24.145	-	-	-	-
38 *	Globulol	1590	51371-47-2	-	-	-	-	0.529	24.208
39 *	*trans*-β-Elemenone	1593	30824-86-3	-	-	-	-	0.135	24.276
40	Curzerenone	1593	20493-56-5	26.078	24.481	-	-	39.937	24.481
41 *	β-Atlantol	1607	38142-56-2	-	-	-	-	0.892	24.606
42	Citronellyl pentanoata	1607	7540-53-6	1.501	24.616	-	-	-	-
43	Syringaldehyde	1601	134-96-3	-	-	0.035	24.843	-	-
44 *	*epi*-Cedrol	1619	199003-73-2	-	-	-	-	1.009	24.871
45	Khusimone	1619	30557-76-7	0.356	24.876	-	-	-	-
46 *	Isolongifolan-7-α-ol	1622	57566-26-4	-	-	-	-	0.831	24.949
47	Isolongifolanone	1629	26839-51-0	0.600	24.962	-	-	-	-
48	Torreyol (α-Muurolol)	1627	19435-97-3	0.453	25.058	-	-	0.316	25.436
49 *	10-*epi*-γ-Eudesmol	1624	15051-81-7	-	-	-	-	1.194	25.064
50	γ-Eudesmol	1632	1209-71-8	3.948	25.171	-	-	2.484	25.189
51	2-Hydroxydiphenyl methane	1632	28994-41-4	-	-	0.102	25.192	-	-
52 *	*allo*-Aromadendrene epoxide	1638	85160-81-2	-	-	-	-	0.341	25.305
53 *	Hinesol	1640	23811-08-7	-	-	-	-	0.449	25.367
54	Cubenol	1641	21284-22-0	0.443	25.374	-	-	-	-
55	α-Muurold	1643	19435-97-3	1.037	25.434	-	-	-	-
56	Desmethoxy encecalin	1648 (CR), 1649 (HP)	10913-07-1	0.817	25.544	-	-	0.749	25.549
57	β-Eudesmol	1655 (SR), 1656 (HP)	473-15-4	-	-	0.091	25.704	3.402	25.709
58	α-Eudesmol	1656	473-16-5	3.310	25.717	-	-	-	-
59 *	Selin-11-en-4-α-ol	1659	16641-47-7	-	-	-	-	1.675	25.784
60	*neo*-Intermeded	1659	5945-72-2	0.478	25.788	-	-	-	-
61	*ar*-Turmerone	1661	532-65-0	-	-	0.099	25.836	-	-
62 *	Germacra-4(15),5,10(14)-trien-1-α-ol	1668	81968-62-9	-	-	-	-	0.488	25.909
63	5-Hydroxy isobornyl isobutanoata	1664	107783-33-5	0.493	25.909	-	-	-	-
64 *	14-Hydoxy-9-*epi*-(*E*)-Caryophyllene	1669	79768-23-5	-	-	-	-	0.679	26.002
65	Cadalene	1672	483-78-3	0.639	26.078	-	-	-	-
66	Germacrone	1693	6902-91-6	2.778	26.560	-	-	4.822	26.563
67 *	Zizanal	1689	82509-29-3	-	-	-	-	0.235	26.665
68	Sinensal	1699	60066-88-8	0.382	26.686	-	-	-	-
69	(*Z*)-β-Santalol	1711	77-42-9	2.550	26.996	-	-	-	-
70 *	Mayurone	1711	4677-90-1	-	-	-	-	0.560	26.961
71	Curcumenol	1720	18431-84-6	0.915	27.142	-	-	-	-
72 *	Longifold	1722	469-27-2	-	-	-	-	0.498	27.183
73	Isobicyclogermacrenal	1723	73256-82-3	0.438	27.196	-	-	-	-
74	Cryptomerione	1729	5988-72-7	1.730	27.422	-	-	-	-
75	Ambroxide	1740 (CR), 1733 (HP)	6970-58-5	1.597	27.575	-	-	0.374	27.415
76 *	Cyclocolorenone	1740	489-45-2	-	-	-	-	0.354	27.568
77	β-Acoradienol	1761	149496-35-5	0.991	28.015	-	-	-	-
78 *	Squamuloseone	1771	34413-94-0	-	-	-	-	0.856	28.396
79 *	14-Hydroxy-α-Muurolene	1781	135118-51-3	-	-	-	-	0.856	28.396
80	(2*E*,6*E*)-Methyl farnesoate	1783	3675-00-1	1.643	28.498	-	-	-	-
81 *	Vetivenic acid	1792	16203-25-1	-	-	-	-	1.287	28.687
82	8-Cedren-13-ol accetate	1792	18319-41-0	2.781	28.695	-	-	-	-
83	1-Octadecene	1794	112-88-9	-	-	0.028	28.727	-	-
84	14-Hydroxy-δ-Cadinene	1802	135118-52-4	0.509	28.925	-	-	1.328	28.84
85 *	2-Ethylhexyl-Salicyate	1810	118-60-5	-	-	-	-	0.314	29.073
86	*iso*-Acorone	1811	6168-64-5	0.396	29.083	-	-	-	-
87	Acorone	1816	10121-28-5	0.405	29.210	-	-	-	-
88 *	Eudesm-7(11)-en-4-ol acetate	1831	67987-89-7	-	-	-	-	0.417	29.499
89	Cyclopentadecanolide	1834	106-02-5	-	-	0.348	29.556	-	-
90	2,7(14),10-Bisabolatrien-1-ol-4-one	1837	216372-16-6	1.090	29.628	-	-	-	-
91 *	Phenyl ethyl octanoate	1838	5457-70-5	-	-	-	-	0.658	29.637
92	*n*-Hexadecanol	1840	36653-82-4			0.476	29.678	-	-
93	(*E*)-Nerolidy isobutyrate	1844	000-00-0	0.504	29.772	-	-	-	-
94	1,10-β-epoxy-6-Oxofuranoeyemophilane	1850	5974-12-0	1.224	29.887	-	-	0.962	29.880
95	Cubitene	1874 (CR), 1864 (HP)	66723-19-1	0.396	30.380	-	-	0.451	30.181
96	*cis*-Thujopsenic acid	1865	546-53-2	1.015	30.199	-	-	-	-
97 *	Dihydro-Columellarin	1905	66873-38-9	-	-	-	-	0.652	30.940
98	Totarene	1903	000-00-0	0.881	30.952	-	-	-	-
99	Isopimara-9(11),15-dien	1906	39702-28-8	0.432	31.021	-	-	-	-
100 *	11,12-dihydroxy Valencene	1919	000-00-0	-	-	-	-	0.155	31.281
101	Methyl hexadecanoate	1923	112-39-0	-	-	0.287	31.351	-	-
102 *	(3*E*)-Cembrene A	1929	31570-39-5	-	-	-	-	0.121	31.481
103	Cyclohexadecanolide	1937	109-29-5	-	-	1.208	31.636	-	-
104 *	Columellarin	1949	66873-37-8	-	-	-	-	0.435	31.860
105	Hexadecanoic acid	1967 (SR), 1955 (HP)	57-10-3	-	-	38.634	32.214	0.266	31.990
106	(3*Z*)-Cembrene A	1964	71213-92-8	1.026	32.162	-	-	-	-
107	Nootkatin	1985	4431-03-2	1.775	32.573	-	-	-	-
108 *	*epi*-Catalponol	1988	70368-23-9	-	-	-	-	1.348	32.625
109	Ethyl hexadecanoate	1991	628-97-7	-	-	1.828	32.687	-	-
110	Polygodial	2014	6754-20-7	0.285	33.117	-	-	0.523	32.851
111	Dolabradiene	2000	134507-28-1	0.747	32.858	-	-	-	-
112 *	Phyllocladene	2015	20070-61-5	-	-	-	-	0.254	33.162
113	Warburganal	2016	62994-47-2	0.296	33.354	-	-	-	-
114	Kaurene	2035	34424-57-2	0.714	33.526	-	-	-	-
115 *	Osthole	2040	484-12-8	-	-	-	-	0.551	33.607
116 *	Sclaredide	2070	564-20-5	-	-	-	-	0.140	34.185
117	Methyl linoleate	2089 (SR), 2124 (HP)	112-63-0	-	-	0.572	34.523	0.099	35.160
118	Nootkatinol	2095	2492-08-2	0.448	34.638	-	-	0.359	34.636
119	*n*-Octadecanol	2102	112-92-5	-	-	0.404	34.763	-	-
120 *	(*E*)-Isoeugenyl Benzyl ether	2116	92666-21-2	-	-	-	-	0.188	35.010
121	Linoleic acid	2124 (CR), 2285 (SR)	60-33-3	0.561	35.531	37.981	35.329	-	-
122 *	Oroselone	2131	1760-27-6	-	-	-	-	0.113	35.279
123 *	Abienol	2151	1616-86-0	-	-	-	-	0.112	35.662
124	*n*-Docosene	2163	629-97-0	-	-	3.196	35.874	-	-
125	1-Docosene	2170	1599-67-3	-	-	0.770	35.983	-	-
Total				75.416		91.857		79.553	

RI refers to the retention index experimentally calculated using C7–C40 alkanes; φ is relative content; t is retention time; CR: *Curcumae Rhizoma*; SR: *Sparganii Rhizoma*; HP: herb pair CR–SR; -: Not detected; * New constituents in herb pair CR–SR.

#### 2.1.1. The Common Volatile Oils of HP and CR

Several characteristics about the volatile chemical components in HP CR–SR can be seen from Table 1. The main volatile chemical compounds in HP were curzerenone (39.937%), 4-hydroxy-4-methyl-2-pentanone (4.343%), germacrone (4.822%), β-eudesmol (3.402%), γ-eudesmol (2.484%), selin-11-en-4-α-ol (1.675%), *epi*-catalponol (1.348%), 14-hydroxy-δ-cadinene (1.328%), and vetivenic acid (1.287%). Thereinto, curzerenone, γ-eudesmol, and germacrone, as the common volatile oils of HP and CR, were also the higher content components of CR, accounting for 26.078%, 3.948%, and 2.778% respectively.

It is noteworthy that curcumenol has broad-spectrum anticancer activity [21] and its content in HP oil was approximately two times higher than that of CR oil. Clinically, germacrone, as the official quality control marker for Zedoary turmeric oil, exhibited many pharmacological activities including anti-tumor activity, antiviral activity, and so on [28,29]. Comparing with CR oil, the content of germacrone was increased in HP oil. Three common components in volatile oils of HP and SR were detected, including 4-hydroxy-4-methyl-2-pentanone (5.691%) and two other trace amount ingredients. The conclusion from the analysis above is that the volatile chemical components in HP CR–SR were not completely the same as those of single herb CR or SR.

#### 2.1.2. The New Chemical Components of HP Volatile Oil

There are 38 terpene alcohol-type components that were first found in HP, such as *epi*-cedrol (1.009%), 10-*epi*-γ-eudesmol (1.194%), selin-11-en-4-α-ol (1.675%), *epi*-catalponol (1.348%), vetivenic acid (1.287%), and so on. Those new component contents in HP reach up to 19.380%. The reports about their pharmacological activities are rather rare. The following compounds were worth mentioning: *epi*-cedrol, as a sesquiterpene alcohol, was also detected in *Artemisia annua* L, which could increase production of artemisinin by genetically-engineered methods [30]. Selin-11-en-4-α-ol was the principle component in the oil of *Ononis Sicula* Guss., exhibited moderate antioxidant activity [31]. It is the purportedly unique sesquiterpene compound, 10-*epi*-γ-eudesmol, that has been suggested as a marker for distinguishing the various cultivars and hybrids of *Pelargonium graveolens* [32]. In addition, vetivenic acid was isolated by column chromatography and identified by NMR and CG-MS from *Vetiveria zizanioides* root, with promising antimicrobial effects [33]. Interestingly, 2-ethylhexyl-salicyate, a significant sunscreen, was widely used in cosmetics and the medicine industry [34]. Mayurone was first reported from *Mayur pankhi* and *Thujopsis dolabrata*, respectively, in 1965, and widely existed in many kinds of plants such as *Panax ginseng* and *Franco Thujaorientalis* [35]. Considering the relatively high content and potential activity of those new components, it is inferred that they may impact on the activity and efficacy of HP oil.

Moreover, some volatile chemical compounds of CR, such as α-eudesmol (3.310%, 8-cedren-13-ol accetate (2.781%), (*Z*)-β-santalol (2.550%), nootratin (1.775%), and cryptomerione (1.730%), have not been detected in HP. In addition, hexadecanoic acid (38.634%), linoleic acid (37.981%), and *n*-docosene (3.196%), as the main chemical ingredients of SR volatile oil, have not been seen in HP. Possible reasons could be listed as follows: for one thing, some organic acids of SR, like succinic acid or sanleng acid, could play a role of solubilization effect, which have improved the constituents of CR dissolving out during the process of decocting the two single herbs, CR and SR [36]. For another, it has a chance to easily interact among many active organic compounds, such as aldehydes, ketones, and alcohols, during the process of decocting CR and SR [3,18,37,38,39,40]. In addition, the total contents of essential oils in CR and SR were reported to be quite uneven (2% and 0.04%, respectively) [36], thus, it probably means that volatile oil of SR were covered by the volatile oil of CR when the compatibility ratio of two herbs is one to one.

### 2.2. DPPH Radical Scavenging Activity

**Figure 1:**
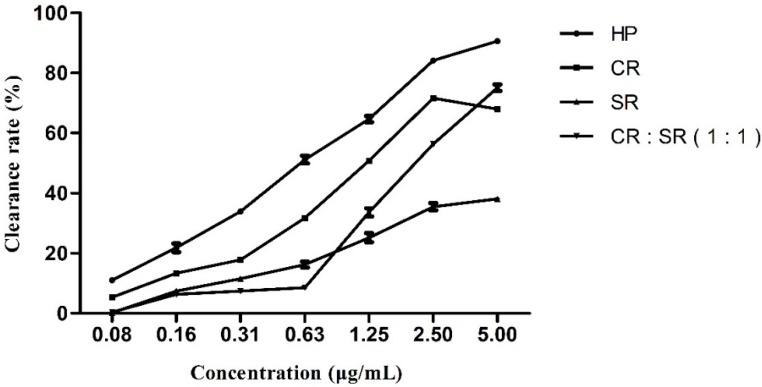
The DPPH free radical scavenging activities of essential oil from CR, SR, and HP CR–SR.

Free radicals and oxidants could affect the metabolism of the body [41]. Antioxidants have become important auxiliary products for health. DPPH is a useful reagent for investigating the free radical scavenging ability of compounds. The dose-response curve of DPPH radical scavenging activities of the essential oils from CR, SR, and HP CR–SR was distinct in Figure 1. The essential oil from HP CR–SR possessed the best activity with a rate of 90.6% under 5.0 mg/mL, and the essential oil from SR showed little DPPH radical scavenging activity.

Among total essential oils of the four samples, HP CR–SR possessed the highest antioxidant activity (IC_50_ = 0.59 ± 0.04 mg/mL). Compared with HP CR–SR oil, the volatile oil of CR showed moderate DPPH scavenging ability (IC_50_ = 1.39 ± 0.06 mg/mL), and the other two samples, SR and CR + SR, their IC_50_ were 8.39 ± 1.20 mg/mL and 2.20 ± 0.09 mg/mL, respectively. In addition, as a positive control, IC_50_ of vitamin C was 0.07 mg/mL in this condition. 

In daily life, the increased dietary intake of natural antioxidants could reduce cancer mortality [42,43,44]. Moreover, antioxidants not only selectively inhibit the proliferation of cancer cells but also do not harm normal cells [45]. This means that the positive correlation exists between the antioxidant and antitumor activities.

### 2.3. Anti-Tumor Activity

The essential oils (CR, SR, and HP) were tested *in* *vitro* for their potential human tumor cell growth inhibitory effect on five human tumor cell lines (BGC823, A549, MCF-7, HeLa, and SKOV3), using MTT assay. The five human tumor cell lines are the most conventional tumor cell lines for the screening of antitumor activity, which are deemed to be high in veracity and reliability.

**Figure 2:**
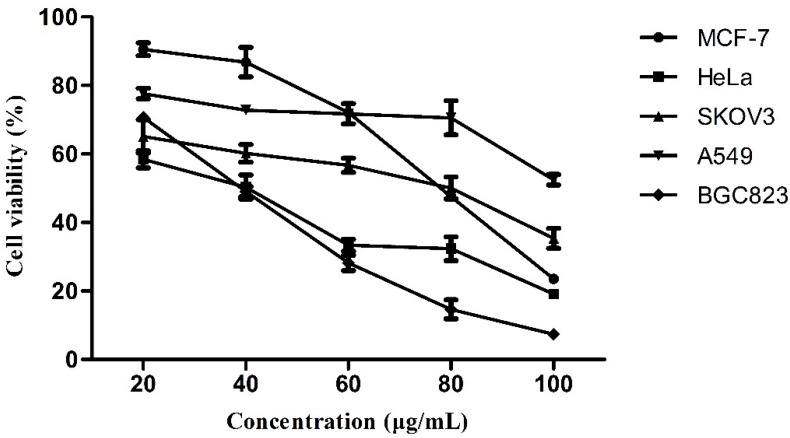
Effect of the HP essential oil on cell viability of five cancer cell lines. Cells were treated with vehicle alone (0.5% DMSO) and specified concentrations of the essential oil in 0.5% DMSO for 24 h; cell viability was determined by the MTT assay as detailed in Section 2. The values are represented as the percent of viable cells, with vehicle-treated cells regarded as 100% viable. Each point represents the mean of the values obtained from three independent experiments.

The concentration that inhibited cell vitality by 50% (IC_50_) is presented in Table 2. Apparently, HP essential oil was the most effective in all tested cell lines, and presented IC_50_ values ranging from 32.32–74.84 μg/mL, except for human lung cancer cell lines (A549). The CR oil had less activity compared with HP oil, IC_50_ in the range between 31.57 and 162.87 μg/mL. The oil of SR had the lowest antitumor activity, and it showed nearly no efficacy in inhibiting, instead promoting five human tumors cell lines. On the other hand, the HeLa cell line was the most susceptible to the oils of CR and HP, while, among the tumor cell lines, the A549 was the most resistant one (Figure 2).

In particular, in order to further compare the activity differences between HP and two single herbs (CR and SR), group (CR + SR) is also set in the test. Different from group HP, the group (CR + SR) was made of the oils of CR and SR with 1:1 input quantity. Seeing from Table 2, IC_50_ values of group HP are all lower than that of group (CR + SR) on human tumor cell growth inhibitory effect. Based on these findings, it can be speculated that the HP oil was remarkably better than the oil of any single one on anticancer activity.

**Table 2:** *In* *vitro* cytotoxic activities of the essential oils.

Treatments	MCF-7 IC_50_ (μg/mL) ^a^	HeLa	SKOV3	BGC823	A549
HP	74.84 ± 1.66	32.32 ± 5.31	66.12 ± 11.23	34.76 ± 1.82	708.24 ± 943.91
CR + SR	184.51 ± 59.09	80.88 ± 10.06	131.02 ± 35.83	48.83 ± 2.76	>1000
SR	>1000	185.19 ± 187.88	>1000	>1000	>1000
CR	82.30 ± 5.36	31.57 ± 8.13	72.23 ± 5.20	44.11 ± 1.95	162.87 ± 43.36

^a^ Results are expressed as IC_50_ values (μg/mL) from three independent experiments performed in quadruplicate, measured by MTT assay after 24 h of incubation. The IC_50_ value, relative to untreated control, represents the concentration that inhibited cell vitality by 50%.

In the inhibitory effect assays on five tested human tumor cell lines, the essential oil of HP, in a dose-dependent manner, was the most effective compared to CR and SR oils. In other words, with the SR being put into HP, more effective ingredients in CR were dissolved out. This is highly consistent with chemical research results, especially being related with new components in HP oil. In spite of having found new constituents in HP oil, their pharmacological activities were less reported. Their activities should be focused on in the further studies.

## 3. Experimental Section 

### 3.1. Materials

Single herb CR and SR, with vinegar processing, were purchased from Hebei Anguo Medical Materials Corporation (Anguo, China), and identified as *Curcuma* *kwangsiensis* S.G. Lee et C.F. Liang and *Sparganium stoloniferum* Buch.-Ham., respectively, by Yuan Zhang from the Beijing University of Chinese Medicine, China. *n*-alkane standard solutions of C7–C40 (No. 49452-U) was purchased from SUPELCO (Bellefonte, PA, USA). 

### 3.2. Extraction of the Essential Oils

A total of 100 g of CR and 100 g of SR single herbs were mixed and soaked with 1000 mL deionized water in a standard extractor for extracting volatile oil at room temperature for 12 h. Then, the essential oil was prepared by using the standard steam distillation method, according to the Chinese Pharmacopeia [9]. The essential oil was collected and stored in a refrigerator at 4 °C before use. The essential oils of single herb CR or SR was extracted in the same method.

### 3.3. GC-MS Analysis

Volatile oils were analyzed by an Agilent Technologies 5975C gas chromatograph (GC) equipped with a HP-5 MS capillary column (5% phenyl methyl Siloxane, 30 m × 0.25 mm i.d., 0.25 μm film thickness) and an HP 5973 mass selective detector (Agilent Technologies, Palo Alto, CA, USA) in the electron impact ionization mode (70 eV) under the following operating conditions: split ratio, 1:40; injection volume, 1.0 µL (TBME solution); inlet temperature, 260 °C; detector temperature, 260 °C; The oven temperature was programmed to start at 50 °C, increased at 5 °C/min to 100 °C, 100 °C increased at 3 °C/min to 150 °C, 150 °C increased at 5 °C/min to 250 °C, then held at 250 °C for 30 min. The helium (99.999%) carrier gas was kept at a constant flow of 1.2 mL/min. 

### 3.4. Identification of Compounds

The chemical ingredients of volatile oils were identified by the retention indices (RI), relative to a series of alkanes (C7–C40) at the same chromatographic conditions, consulting the Van Den Dool method [46]. The data were analyzed by the Xcalibur 1.1 software (Thermo Fisher Scientific, Waltham, MA, USA, version 1.1), compared with the NIST/EPA/NIH database (2005, version 2.0d, Scientific Instrument Services, Inc., Ringoes, NJ, USA) and several references [47,48,49,50]. The book “*Identification of Essential Oils Components by Gas Chromatogrtaphy*/*Mass Spectrometry*, 4th edition” [51] played an equally vital role in identifying the individual compounds.

### 3.5. Anti-Tumor Activity

Human gastric cancer cell lines (BGC823), human lung cancer cell lines (A549), human breast carcinoma cell lines (MCF-7), human cervix carcinoma cell lines (HeLa), and human ovary carcinoma cell lines (SKOV3) were purchased from Cell Resource Center, Institute of Basic Medical Sciences, Chinese Academy of Medical Sciences and Peking Union Medical College, Beijing, China. These cell lines were maintained in RPMI 1640 culture medium plus 10% calf serum and 100 U/mL penicillin, 75 U/mL streptomycin, in a 37 °C incubator supplied with 95% room air and 5% CO_2_. 

The growth inhibitory effect of essential oils of HP, CR, and SR were measured using the standard MTT assay [52,53]. Briefly, 100 μL samples of cell suspension (6 × 10^4^ cells/well) were cultivated in 96-cell plates for 18 h, as described. The samples of HP, CR, and SR oil were well dissolved in DMSO, respectively, followed ultrasonic vibration. Then, cells were incubated for 24 h with varying concentrations of the oil. After adding 100 μL MTT (1 mg/mL) solution to each well, the cells were further incubated at 37 °C for15 min, in a humidified atmosphere of 95% air/5% CO_2_. For MTT assay, the supernatant was thrown away and 200 μL DMSO was mixed into, the 96 wells plate was oscillated on micro-vibrator for an additional 10 min, the absorbency of each well was determined at λ 560 nm by an enzyme-immunoassay instrument (Beijing Perlong New Technology Co., Ltd., Beijing, China). Anti-tumor activity was expressed as the concentration of the drug inhibiting cell growth by 50% (IC_50_ value).

### 3.6. DPPH Radical Scavenging Activity

The DPPH radical scavenging activity was determined according to the method reported by Sharififar and others with some modifications, and compared with the activity of vitamin C as a positive control [54]. An equal amount of (1 mL of 0.05 mg) DPPH solution in ethanol was mixed with 0.1 mL essential oil from CR, SR, and HP CR–SR in ethanol at various concentrations. After mixing, the solution was allowed to reach a steady state at room temperature for about 30 min. The DPPH radical scavenging activity was determined by the absorbance at λ 517 nm with the DNM9602A microplate reader (Beijing Perlong New Technology Co., Ltd., Beijing, China) and calculated by the following equation:

DPPH radical scavenging activity = (A_0_ − A_1_)/A_0_ × 100%

where A_0_ is the absorbance of the control (blank, without extract) and A_1_ is the absorbance of the mixture with the essential oil. 

## 4. Conclusions

In the present study, it is first reported that the essential oils of CR, SR, and HP CR–SR were comparatively explored from the aspects of the chemical compositions, antioxidant, and anticancer activities. The essential oil of HP is different from any, one of CR and SR, nor simply their superposition. Thirty-eight new components were detected in HP, and the relative contents of those new components reach up to 19.380%. Moreover, HP oil showed significant antioxidant and anticancer activities and the essential oils from four groups on antioxidant and anticancer activities were consistent, in order: SR < CR + SR < CR < HP. It is well known that the essential oil of CR reveals prominent activities in the clinic, and it has been documented in different versions of Chinese Pharmacopoeia since 1977. Compared with CR oil, HP oil possessed the stronger antioxidant and anticancer activities. Thus, the volatile oil of HP has the better medicinal application prospect and deserves more attention in the pharmaceutical industry.

HP CR–SR belongs to a mutual reinforcement relationship according to seven compatibilities in TCM theory. Comparison and analysis of the essential oils of CR, SR, and HP CR–SR from chemical compositions and pharmacological activities, we can infer that CR may play a predominant role in HP, namely sovereign drug according to TCM theory. In spite of the low concentration of SR oil, it also plays an important part in HP through improving the dissolution of effective constituents and helping interact to produce new ingredients. In order to further illustrate the role of SR in HP, more focus can be concentrated on the change of HP essential oils (produced from different proportion compatibilities of CR and SR) on the chemical composition and pharmacological activities. Furthermore, it can be concluded that the chemical composition in HP CR–SR is responsible for its potent anticancer and antioxidant activities. Further investigations are in progress in our laboratory to identity the exact active principle and their action mechanism involved in anticancer and antioxidant activity. 

Recently, the studies about herb pairs mostly concentrated on the content change of some main components and traced the origin of chemical constituents [55,56,57,58]. Based on the integrity of TCM theory, it is suggested to be more focused on the content change of common components and the appearance of new constituents in herb pairs with different proportion compatibilities. Such experimental design and research approaches are encouraged to be applied in the further study on HP CR–SR decoction, in order to clarify the compatibility essence of HP.
